# Effect of Organoclay Content on the Physicochemical and Separation Properties of PVDF/Clay Nanocomposite Membranes

**DOI:** 10.3390/polym18121424

**Published:** 2026-06-07

**Authors:** Jun Zhang, Boming Fan, Fengmei Shi, Chao Lin, Shuqi Ma, Qi Shen, Jinglong Yuan, Hua Fan, Yuxin Ma

**Affiliations:** 1 College of Civil Engineering, Heilongjiang University, Harbin 150080, China; zhangjun@hlju.edu.cn (J.Z.);; 2 Heilongjiang Academy of Black Soil Conservation and Utilization, Heilongjiang Academy of Agricultural Sciences, Harbin 150086, China; ocean-water@126.com; 3 College of Airspace Engineering, Harbin Engineering University, Harbin 150001, China; 4 Research and Development Centre, Shandong Aisen Water Industry Co., Ltd., Taian 271021, China

**Keywords:** antifouling, hydrophilicity, protein rejection, ultrafiltration, nanocomposite membrane, organoclay

## Abstract

Polyvinylidene fluoride (PVDF)/clay nanocomposite membranes with different nanoclay loadings (0–5 wt%) were prepared via the non-solvent induced phase inversion method. Effects of organo-montmorillonite (OMMT) content on the morphology and performance were systematically investigated. Results showed that OMMT was uniformly exfoliated and dispersed in the PVDF matrix, while the nanocomposite membranes consistently maintained the β-crystalline phase of PVDF. The incorporation of nano-clay significantly enhanced membrane hydrophilicity, porosity, pure water flux, and protein rejection performance: when clay content increased to 5 wt%, the pure water flux improved from 89.8 L·m^−2^·h^−1^ to 216.3 L·m^−2^·h^−1^, with rejection rates of 98.6% for bovine serum albumin (BSA) and 95.1% for pepsin. Mechanical tests showed that 3 wt% was the optimal clay loading, at which the storage modulus of the membrane increased by 59.8% compared to neat PVDF membranes. Antifouling experiments revealed that the nanocomposite membranes exhibited significantly lower irreversible fouling resistance, substantially improved hydraulic cleaning flux recovery rates, and markedly enhanced antifouling properties. Furthermore, long-term stability, economic analysis, and environmental safety assessments confirmed the practical application potential of these nanocomposite membranes in water treatment. These findings provide theoretical support and technical references for the preparation and application of high-performance PVDF ultrafiltration membranes.

## 1. Introduction

Polyvinylidene fluoride (PVDF), as an important membrane material, has broad application prospects in the field of water treatment due to its excellent chemical stability, thermal stability, and mechanical strength [1]. PVDF membranes are widely manufactured as flat-sheet, hollow fiber, and spiral-wound modules, among which flat-sheet and hollow fiber configurations are most commonly adopted in industrial applications because of their high packing density, convenient assembly, cleaning, and replacement. In terms of separation processes, PVDF membranes are predominantly applied in ultrafiltration (UF) and microfiltration (MF) for drinking water purification, industrial wastewater treatment, protein separation, and resource recovery [2,3,4]. Commercial PVDF UF/MF membranes are widely used owing to their stable performance under harsh operating conditions; however, the strong inherent hydrophobicity of PVDF—typically with a water contact angle of 80°~90°—leads to severe membrane fouling caused by the adsorption of proteins, natural organic matter, and colloidal pollutants [2,5]. Such fouling leads to rapid flux decline, higher energy consumption, more frequent chemical cleaning, and shortened service life, which greatly restricts the long-term and high-efficiency application of PVDF membranes [2,6].

To address the hydrophobicity-induced fouling issue, researchers have developed various modification methods to enhance the hydrophilicity of PVDF membranes, including surface coating, chemical grafting, and blending with hydrophilic additives [7,8,9]. Among these approaches, incorporating inorganic nanomaterials into polymer matrices to fabricate mixed matrix membranes (MMMs) is considered an efficient and feasible modification strategy [2,3,4,8,9,10,11,12,13,14,15]. Nanoparticles such as TiO_2_ [4,8,9], Al_2_O_3_ [10], SiO_2_ [11,15], emerging sustainable nanocellulose-based materials, such as cellulose nanocrystals (CNC) [12] and cellulose nanofibers (CNF) [13], and carbon-based nanomaterials represented by carbon nanotubes (CNTs) [14] have been extensively studied for PVDF membrane modification, as they can enhance hydrophilicity and antifouling properties to a certain extent. These diverse nanomaterials endow PVDF nanocomposite membranes with tunable structures and upgraded separation performance for water treatment applications. However, these nanoparticles commonly face challenges like agglomeration in the polymer matrix and potential leakage during long-term operation, which compromise membrane stability and separation performance [16].

Despite TiO_2_, SiO_2_, and other nanoparticle modifications having made progress, the layered structure of clay materials endows them with greater potential in dispersibility and performance enhancement [17,18]. Clay materials, especially organically modified montmorillonite (OMMT), which are naturally abundant, low-cost, eco-friendly, and sustainable, have attracted increasing attention due to their unique layered structure, high aspect ratio, excellent dispersibility, and ability to synergistically improve membrane hydrophilicity, mechanical strength, and antifouling properties [18,19]. Montmorillonite (MMT) is a natural layered silicate with weak van der Waals forces between layers, leading to poor dispersion in polymer matrices and weak interactions with polymer chains. OMMT is obtained by modifying MMT with organic surfactants (e.g., quaternary ammonium salts), which increases the interlayer spacing, improves compatibility with polymer molecules, and enables exfoliated dispersion in the polymer matrix, forming a uniformly dispersed structure that strengthens the membrane matrix and reduces compaction [18].

Although existing studies on PVDF/clay nanocomposite membranes have laid a foundation, several critical gaps remain. Li and Kim [17] found that modified montmorillonite improved the thermal degradation resistance of nanocomposite membranes but did not investigate transport performance and fouling characteristics. Hwang et al. [18] demonstrated that nanoclay enhanced the mechanical strength of PVDF porous supports but paid limited attention to separation efficiency. Rajabi et al. [19] demonstrated that OMMT incorporation improved membrane hydrophilicity, pure water flux, and antifouling performance. However, their study focused on a limited set of performance indicators and did not provide a comprehensive investigation of crystalline structure, mechanical properties, long-term stability, environmental safety, and economic feasibility, which are essential for evaluating the overall performance and practical potential of nanocomposite membranes. Moreover, unlike amorphous polymers such as polysulfone (PSf), PVDF is a semi-crystalline polymer, and the interaction mechanism between OMMT and PVDF crystal structure, as well as the evolution law of nanocomposite membrane structure and performance with clay content, remains unclear [20].

To fill these research gaps, flat-sheet PVDF/OMMT nanocomposite ultrafiltration membranes with 0–5 wt% clay loading were successfully fabricated via the non-solvent phase inversion (NIPS) method. The novelties and superiorities of this study are highlighted as follows: (1) OMMT achieves uniform and complete exfoliation in the PVDF matrix over the entire loading range, overcoming the common agglomeration bottleneck at high filler content. (2) The effects of OMMT content on membrane morphology, crystalline structure, porosity, permeability, protein rejection, thermal stability, mechanical properties, and antifouling behavior are comprehensively investigated, revealing a clear loading-morphology/structure-performance relationship. (3) The mechanism by which exfoliated OMMT stabilizes the dominant β crystalline phase of PVDF is clarified, providing new insights into crystalline regulation of PVDF nanocomposite membranes. (4) The optimal OMMT content (3 wt%) is determined for balanced flux, rejection, mechanical strength, and antifouling performance. (5) Long-term stability, environmental safety, and economic analysis are systematically conducted for the first time, confirming the practical viability of the nanocomposite membranes in real water treatment systems. This study not only reveals the fundamental mechanism of organoclay-PVDF interactions but also provides a scalable, low-cost, and high-performance modification strategy for PVDF ultrafiltration membranes. The findings offer important theoretical guidance and technical support for the development and industrial application of advanced antifouling membranes for sustainable water treatment.

## 2. Materials and Methods

### 2.1. Materials

PVDF (Kureha W#1300) with a number-average molecular weight (Mn) of 350,000 and an inherent viscosity of 1.30 dL/g was purchased from Kureha Chemical Industry Co., Ltd. (Tokyo, Japan). N,N–dimethyl acetamide (DMAc, analytical grade, used as a solvent to dissolve PVDF) and lithium chloride (LiCl, analytical grade, used as a pore-forming agent to increase porosity and accelerate phase inversion) were obtained from Tianjin Bodi Chemicals Co., Ltd. (Tianjin, China) and Beijing Xinguang Chemicals Factory (Beijing, China), respectively. OMMT (BT-880, average particle size 20–50 nm) was obtained from Zhejiang Anji Tianlong Organic Bentonite Co., Ltd. (Anji, China). Bovine serum albumin (BSA, molecular weight *M*_W_ = 68,000 Da) and pepsin (*M*_W_ = 35,000 Da) were acquired from Merck KGaA (Kenilworth, NJ, USA). Deionized water used in all experiments was produced by a laboratory water purification system (resistivity ≥ 18.2 MΩ·cm).

### 2.2. Membrane Preparation

The composition of the casting dope for PVDF/clay nanocomposite membranes is summarized in Table 1. The detailed preparation process was as follows: First, a predetermined amount of OMMT was added to DMAc at room temperature (25 ± 2 °C) and stirred at a constant speed of 200 r/min for 30 min to achieve uniform dispersion; subsequently, PVDF powder and LiCl were added to the OMMT/DMAc dispersion, and the mixture was heated to 60 ± 2 °C with continuous stirring at 200 r/min for 12 h to ensure complete dissolution of PVDF. After dissolution, the casting dope was left at 50 ± 2 °C for at least 24 h to remove the air bubbles generated during stirring. The homogeneous casting solutions were cast uniformly onto a glass substrate by a hand-casting knife with a knife gap set at 200 µm. The cast membrane was immediately immersed in a deionized water coagulation bath (25 ± 2 °C) to conduct NIPS. Being highly water-soluble, LiCl is completely dissolved into the coagulation bath and washing water during NIPS and membrane rinsing. The as-formed membranes were soaked in deionized water for 48 h to ensure full removal of residual LiCl. Subsequently, the prepared membrane was placed in a water bath to rinse away residual solvents and additives, totaling three cycles (each lasting 2 h).

### 2.3. Characterization of Membranes

The membrane morphologies were examined with a scanning electron microscope (SEM). The dispersion of nanoparticles in the membrane was observed with transmission electron microscopy (TEM). Atomic force microscopy (AFM, Bruker Dimension Icon (Santa Barbara, CA, USA)) was used to characterize the surface morphology and roughness of the membranes in tapping mode over an area of 1 μm × 1 μm. The key roughness parameters including arithmetic mean roughness (*R*_a_), root mean square roughness (*R*_q_), and maximum profile height (*R*_t_) were calculated using the instrument software. The permeability, fouling experiments and retention properties of the fabricated membranes were evaluated using a cross-flow filtration experimental apparatus [9,21,22]. All flux measurements were conducted at a transmembrane pressure (TMP) of 100 kPa, a membrane thickness of 200 μm (wet state) at a temperature of 25 °C. The pore size distribution (PSD) was determined using the liquid–liquid displacement method [9]. The membrane surface zeta potential was measured using a Surpass Anton Paar zeta potential analyzer (Graz, Austria) with 0.001 mol/L KCl electrolyte at pH 7.0 ± 0.1. Each sample was tested in triplicate to determine the mean and standard deviation. Membrane porosity was determined using the dry-wet method [9]. The membrane hydrophilicity was characterized with contact angle (CA) measurements. The crystalline structure was characterized by X-ray diffraction (XRD). Chemical structure and crystalline conformation were analyzed by Fourier-transform infrared spectroscopy (FTIR). The thermal properties were tested by thermogravimetric analysis (TGA) and differential scanning calorimetry (DSC); mechanical properties were tested by dynamic mechanical analysis (DMA) and tensile tests. Tensile strength and elongation at break of membranes were determined by a universal electronic strength measurement (AGS-J, Shimadzu (Kyoto, Japan)). All membrane samples were prepared into standard dumbbell shapes, and at least five parallel samples were tested for each group to obtain average values and standard deviations. All of the detailed procedures for all experiments are provided in Supplementary File S1.

### 2.4. Long-Term Stability Test

The PC3 and PC0 membranes showed optimal comprehensive performance and were selected for the long-term stability test with a cross-flow filtration experimental setup (Figure S1). The feed water was the dechlorinated finished water from Harbin Mopanshan Water Treatment Plant, with the following water quality parameters: pH = 7.2 ± 0.05, Al^3+^ = 0.012 ± 0.002 mg/L, Mg^2+^ = 11.32 ± 0.02 mg/L, Ca^2+^ = 14.03 ± 0.02 mg/L, Fe^3+^ = 0.008 ± 0.002 mg/L, and TOC = 0.05 ± 0.01 mg/L. The membrane was operated continuously for 30 days under the following conditions: TMP = 100 kPa, cross-flow velocity = 10 L/h, operating temperature = 25 ± 2 °C. The permeate flux was monitored every 2 days to calculate the flux decline rate.

### 2.5. Environmental Safety Assessment

The dissolution of OMMT components (Al, Ca, Mg, Fe) from the PC3 membrane during the 30-day long-term operation was quantified using inductively coupled plasma mass spectrometry (ICP-MS, Agilent 7900 (Santa Clara, CA, USA)). The total organic carbon (TOC) content and pH of the permeate were measured using a Shimadzu TOC-L CPH analyzer (Kyoto, Japan) and a LICHEN pH-100 pH meter (Shaoxing, China), respectively. The dissolution concentrations were compared with Standards for Drinking Water Quality [23] (GB 5749-2022, China) to evaluate environmental safety.

### 2.6. Economic Estimation

The economic feasibility of the PVDF/clay nanocomposite membranes was evaluated by analyzing the total cost, including raw materials cost, operational cost, and maintenance costs.

Raw material cost: Calculated based on the price of PVDF (¥80/kg), OMMT (¥50/kg), DMAc (¥6/kg), and LiCl (¥200/kg), with the composition listed in Table 1.

Operational cost: Includes membrane replacement cost (based on service life), chemical cleaning cost (citric acid and sodium hypochlorite), and energy consumption (calculated from pump power and operating time).

Maintenance cost: Covers labor and equipment maintenance expenses.

The cost comparison was conducted between the PVDF/clay nanocomposite membrane (PC3) and the neat PVDF membrane (PC0), and the comprehensive operational cost savings rate was calculated. Additionally, the scalability of the membrane preparation process was evaluated by assessing compatibility with existing industrial membrane production lines.

## 3. Results and Discussion

### 3.1. Morphological Study

**Figure 1:**
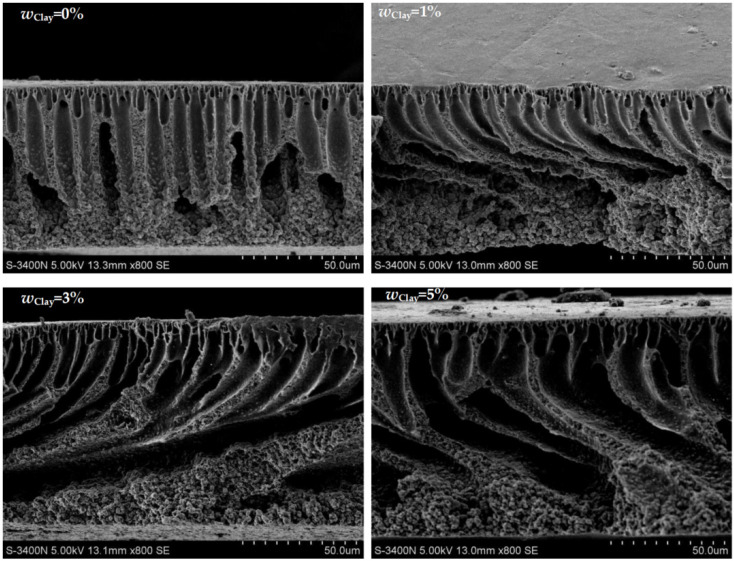
Cross-section SEM images of PVDF/clay nanocomposite membranes with different *w*_Clay_.

Figure 1 shows the cross-sectional SEM images of PVDF/clay nanocomposite membranes with different clay contents. As shown in the cross-sectional SEM images (Figure 1), the total membrane thickness increases with the OMMT content. All membranes were cast using a fixed knife gap (200 μm) to ensure identical wet film thickness. This trend can be explained by two factors: (1) First, the addition of OMMT increases the viscosity of the casting solution [20], which affects phase inversion kinetics and contributes to maintaining a thicker structure. (2) Secondly, the rigid OMMT platelets restrict the shrinkage of the PVDF matrix during solvent evaporation and drying processes, thereby improving dimensional stability. The combined effects of increased casting solution viscosity and reduced shrinkage result in greater final dry thickness for the nanocomposite membranes. It is also observed that all membranes exhibit a typical asymmetric structure composed of the dense skin layer, the intermediate transitional layer, and the porous support layer. As the clay content increases from 0 to 5 wt%, the finger-like macrovoids in the membrane become significantly larger, pore connectivity improves markedly, overall porosity increases, and the structure becomes more loosely packed. This phenomenon is attributed to the enhanced solvent/non-solvent exchange rate during phase inversion caused by clay incorporation. Simultaneously, clay platelets act as nucleating agents promoting spherulite growth, thereby influencing phase separation kinetics and forming a more developed pore structure [24,25]. The wet membrane thickness was strictly controlled at 200 μm for all samples. The dry membrane thickness of the OMMT-modified membranes was slightly higher than that of the neat PVDF membrane, confirming that OMMT reduces membrane shrinkage during drying and enhances structural stability.

**Figure 2:**
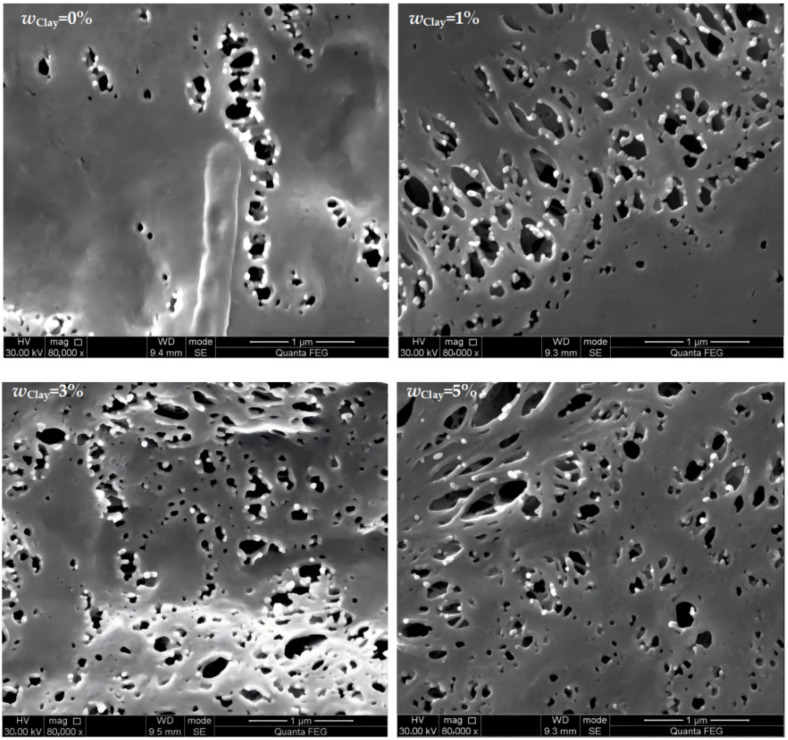
Upper surface SEM images of PVDF/clay nanocomposite membranes with different *w*_clay_.

Figure 2 presents the SEM images of the membrane surfaces with different clay contents. Surface SEM observations show visible differences in surface porosity with increasing OMMT content. However, limited small field-of-view images (≈3 × 3 μm) are insufficient for quantitative conclusions, due to the inherent local heterogeneity of membrane surfaces. Similar small dark features can be observed on both neat and modified membranes.

The improved surface porosity and overall pore connectivity are supported by enhanced pure water flux and more developed cross-sectional structures, rather than localized surface features. With increasing OMMT loading, the membrane surface tends to become more open and permeable, consistent with the overall improvement in transport properties.

**Figure 3:**
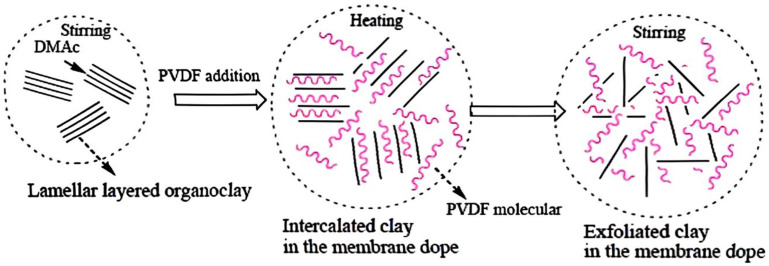
The schematic illustration of clay dispersion in the membrane dope.

Figure 3 shows the schematic diagram of the dispersion process of clay in the membrane casting dope. During membrane preparation, DMAc initially penetrates into the interlayer spaces of organic clay (entropy-decreasing process). When PVDF is added and dissolved, PVDF molecules exchange with DMAc molecules, occupying the interlayer spaces of clay (an entropy-increasing process), which increases the interlayer spacing of clay platelets and forms an intercalated structure. As more PVDF molecules enter the interlayers and under the influence of stirring shear forces, the interactions between platelets further weaken until they disappear, ultimately forming an exfoliated structure. The exfoliated clay platelets are randomly dispersed in the casting solution [20]. These exfoliated clay platelets possess both hydrophilic and hydrophobic groups, which can reduce the water-casting solution interfacial tension and affect the solvent-nonsolvent exchange rate during the phase inversion [20]. During non-solvent-induced phase separation, solvent-nonsolvent exchange triggers liquid–liquid demixing. The distinct finger-like pore structure observed in all membranes is generated by the nucleation and continuous growth of polymer-lean liquid phases, which is a typical feature of binodal phase separation. The incorporated OMMT changes the rheological property of casting dope and phase separation rate, thereby regulating the size and distribution of finger-like pores [24,25].

### 3.2. Crystalline Structure and Clay Dispersion

**Figure 4:**
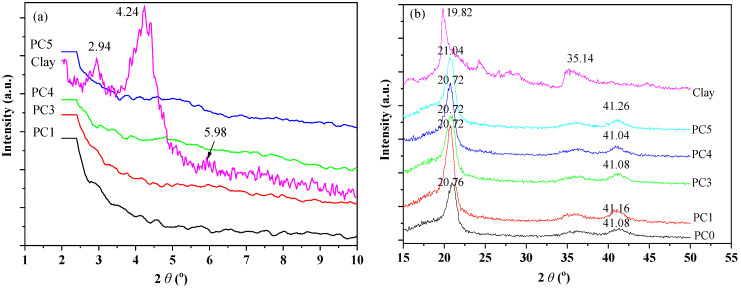
Small-Angle XRD diffractograms (**a**) and XRD diffractograms (**b**) of clay (OMMT), PVDF/clay nanocomposite membranes with various clay contents.

**Figure 5:**
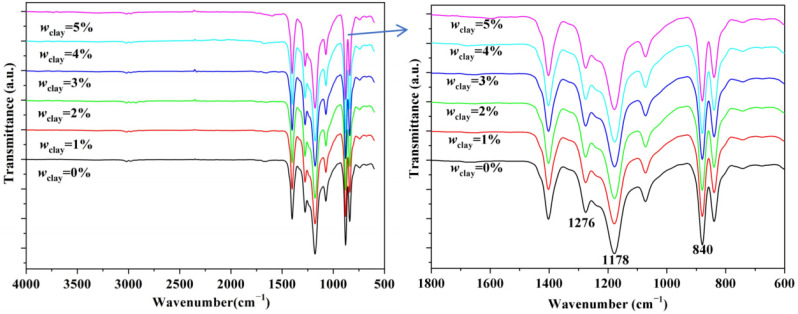
FTIR spectra of PVDF/clay nanocomposite membranes with different *w*_Clay_.

PVDF exhibits five crystalline forms: α, β, γ, δ, and ε, among which the α, β, and γ forms are the most common [26]. The β-form PVDF possesses an all-trans (TTT) planar zigzag conformation, demonstrating piezoelectric properties; the α-form adopts a TGTG/- conformation and is nonpolar; the γ-form has a TTTGTTG/- conformation [27,28]. Figure 4a,b present the Small-Angle XRD diffractograms and the XRD diffractograms of clay (OMMT) and PVDF/clay nanocomposite membranes with various clay contents, which reflect the clay intercalation/exfoliation and PVDF crystallization, respectively. Figure 4a primarily demonstrates the diffraction behavior of the clay substrate, exhibiting sharp and intense peaks at 2θ ≈ 4.24° and 2.94°. The corresponding interlayer spacings are 2.09 nm and 3.00 nm, which correspond to the d_002_ and d_001_ crystal planes, respectively. However, these characteristic peaks are not observed in the XRD patterns of either neat PVDF membranes or nanocomposite membranes, indicating that the clay may have undergone exfoliation or delamination within the PVDF matrix. Figure 4b shows that the crystalline form of the film remains unchanged after clay addition, predominantly exhibiting β-crystal morphology. Figure 5 presents the FTIR spectra of PVDF/clay nanocomposite membranes with different clay contents. Results reveal that all membrane samples exhibit characteristic peaks of the β-form PVDF at 1276 cm^−1^ (CH_2_ wagging vibration), 1178 cm^−1^ (CF_2_ stretching), and 840 cm^−1^ (CF_2_ bending, β form). No characteristic peaks of the α-form (976, 866, 794, 759, and 610 cm^−1^) were detected [29,30,31], suggesting that the nanocomposite membrane consistently maintains the β-crystalline form structure of PVDF, and the addition of clay does not alter the crystalline form of PVDF.

The XRD and FTIR results confirm that all membranes retain a dominant β form of PVDF crystal regardless of OMMT content. OMMT addition does not alter the crystalline modification of PVDF but helps maintain the dominance of the β form during phase inversion.

**Figure 6:**
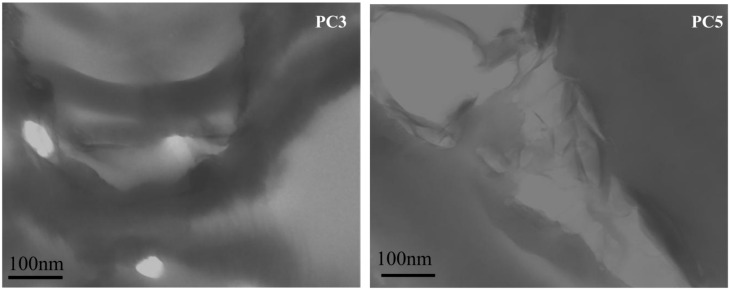
TEM images of PC3 and PC5 nanocomposite membranes.

Figure 6 shows the TEM images of PC3 and PC5 nanocomposite membranes which reflect the clay intercalation/exfoliation. The TEM image reveals that the clay platelets are uniformly dispersed within the PVDF matrix in both PC3 (3 wt%) and PC5 (5 wt%) nanocomposite membranes without significant agglomeration, confirming effective clay dispersion. It should be noted that the disappearance of characteristic clay peaks in XRD patterns cannot serve as sole evidence for exfoliated structures, since factors such as clay dilution, peak broadening, and preferential orientation may interfere with XRD characterization results [32,33]. In contrast, TEM provides morphological, structural, and spatial distribution information about the dispersed phase in nanocomposites at local regions. Therefore, XRD and TEM are complementary techniques for characterizing polymer-clay nanocomposites [34].

The phenomenon where clay acts as a nucleating agent yet leads to reduced crystallinity can be explained as follows: Although clay platelets can serve as heterogeneous nucleation sites to accelerate the crystallization process, their dispersion within the PVDF matrix simultaneously hinders the ordered arrangement of polymer chains and restricts spherulite growth. This competitive effect between nucleation promotion and growth restriction ultimately results in decreased overall crystallinity. Consequently, the crystallinity decreases with increasing clay content due to this antagonistic mechanism.

### 3.3. Surface Roughness and Surface Charge Properties

#### 3.3.1. Surface Roughness (AFM)

**Figure 7:**
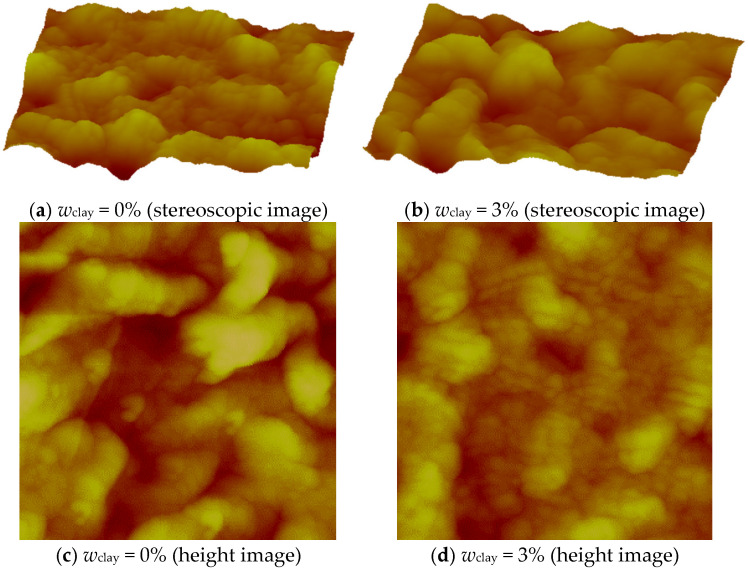
AFM three-dimensional and height images of (**a**,**c**) neat PVDF membrane (*w*_clay_ = 0%) and (**b**,**d**) PVDF/3 wt% OMMT nanocomposite membrane (scan size: 1 μm × 1 μm).

The AFM three-dimensional images and height images of the neat PVDF membrane (*w*_clay_ = 0%) and the optimal PVDF/OMMT nanocomposite membrane (*w*_clay_ = 3%) are presented in Figure 7. The surface roughness values are summarized in Table 2.

The results show that the surface roughness of the membrane significantly increases after the introduction of OMMT. For the neat PVDF membrane, the root mean square roughness (*R*_q_) is 7.51 nm, the arithmetic mean roughness (*R*_a_) is 5.94 nm, and the maximum profile height (*R*_tm_) is 53.8 nm. In contrast, for the membrane with 3 wt% OMMT, *R*_q_ increases to 11.5 nm, *R*_a_ increases to 9.35 nm, and *R*_tm_ rises to 66.4 nm. The surface area difference (ds) also increases from 4.29% to 8.55%.

The increased surface roughness and surface area provide a larger effective water-passing area, which contributes to the improvement of pure water flux. Meanwhile, the uniformly distributed OMMT nanoparticles refine the membrane surface nanostructure, which helps reduce the adsorption and adhesion of protein contaminants.

#### 3.3.2. Zeta Potential

The membrane surface zeta potential values at pH 7.0 are listed in Table 3. The neat PVDF membrane shows a zeta potential of −16.9 ± 0.2 mV, while the 3 wt% OMMT membrane exhibits a more negative value of −25.3 ± 0.3 mV.

The enhanced electronegativity originates from the hydroxyl groups and silicate structures on the exfoliated OMMT layers. Since BSA molecules are negatively charged at neutral pH, the stronger electrostatic repulsion between the membrane surface and protein molecules effectively reduces protein deposition and pore blocking, thereby significantly improving the antifouling performance of the nanocomposite membranes.

### 3.4. Hydrophilicity and Porosity

**Figure 8:**
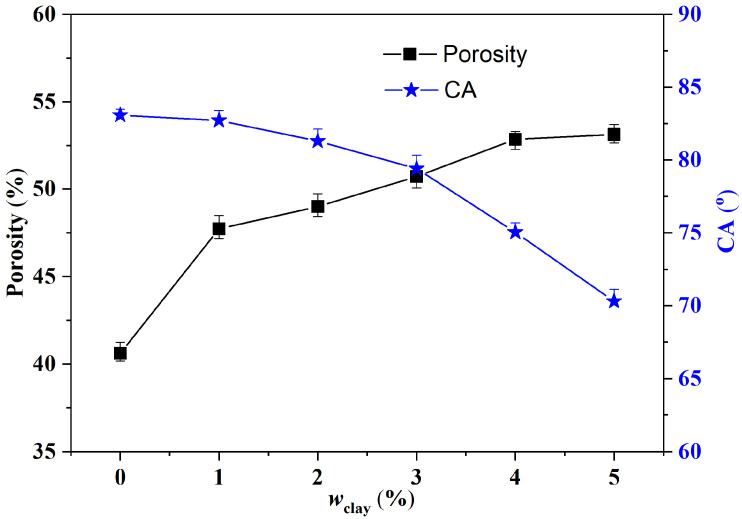
Porosity and contact angle (CA) of PVDF/clay nanocomposite membranes with various *w*_Clay_.

Figure 8 is the porosity and contact angle (CA) of PVDF/clay nanocomposite membranes with various clay contents. The CA and porosity test results show that as the clay content increases from 0 to 5 wt%, the contact angle of the membrane decreases from 83.1° to 70.3°, while the porosity increases from 40.6% to 53.1%. The addition of clay enhances the water inflow rate and restricts the migration of DMAc in the casting solution, thereby increasing the water content in the nascent membrane and improving its porosity. Simultaneously, partially exfoliated clay layers are exposed on the membrane surface, and their hydrophilic groups enhance the hydrophilicity of the membrane. To eliminate the influence of porosity on the contact angle, nanocomposite membranes with different clay contents but identical porosities were prepared by adjusting the porogen content in the casting solution. Test results indicate that even at the same porosity, an increase in clay content significantly reduces the membrane’s contact angle, confirming that the inherent hydrophilicity of clay itself is a key factor in enhancing the hydrophilicity of the membrane surface. The increased porosity and improved pore connectivity with increasing OMMT content can be well supported by previous studies [24,25]. The hydrophilic OMMT nanoparticles accelerate the solvent–nonsolvent exchange rate during the NIPS process, which promotes the formation of a more porous structure. Meanwhile, exfoliated OMMT platelets act as heterogeneous nucleating agents that facilitate the formation of more uniform and abundant pore channels throughout the membrane matrix [20]. Consequently, the porosity increases significantly while the pore structure becomes more developed and interconnected. Such porosity enhancement induced by organoclays during phase inversion has been widely verified in PVDF and other polymeric membrane systems [20].

### 3.5. Membrane Permeability, Solute Rejection and Pore Size Distribution

**Figure 9:**
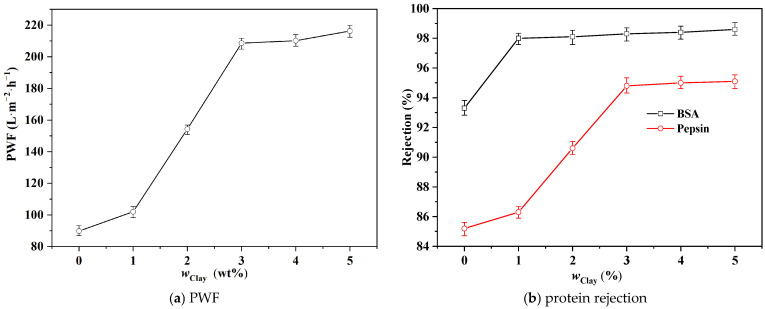
Effect of *w*_Clay_ on the PWF (**a**) and protein rejection (**b**) of PVDF/clay nanocomposite membranes.

Figure 9a is the pure water flux (PWF) of PVDF/clay nanocomposite membranes with various clay contents. The PWF results show that the pure water flux of the neat PVDF membrane (PC0) is 89.8 L·m^−2^·h^−1^. As the clay content increases the PWF gradually increases, reaching 216.3 L·m^−2^·h^−1^ at a clay content of 5 wt% (PC5), which represents an increase of 140.9% compared to the neat PVDF membrane. Figure 9b is the protein rejection rate of PVDF/clay nanocomposite membranes with various clay contents. The protein rejection rate test results indicate that the BSA rejection rate rapidly increases from 93.3% for PC0 to 98.0% for PC1 and then gradually rises to 98.6% for PC5; the pepsin rejection rate increases from 85.2% for PC0 to 94.8% for PC3, followed by a slight increase to 95.1% for PC5. BSA rejection is dominated by size exclusion and electrostatic repulsion: (1) Size exclusion: The average pore size of the PC3 membrane (2.9 nm) is smaller than the hydrodynamic diameter of BSA (4.0 nm), preventing BSA passage; (2) Electrostatic repulsion: The membrane surface is negatively charged (Table 3) at pH 7.0 ± 0.1, while BSA is also negatively charged (isoelectric point ~4.7), leading to electrostatic repulsion that further inhibits BSA adsorption and passage. For pepsin (hydrodynamic diameter 2.8 nm, isoelectric point ~1.0), size exclusion is the main mechanism, and the slight increase in rejection (from 85.2% to 95.1%) is attributed to the refined pore size distribution.

**Figure 10:**
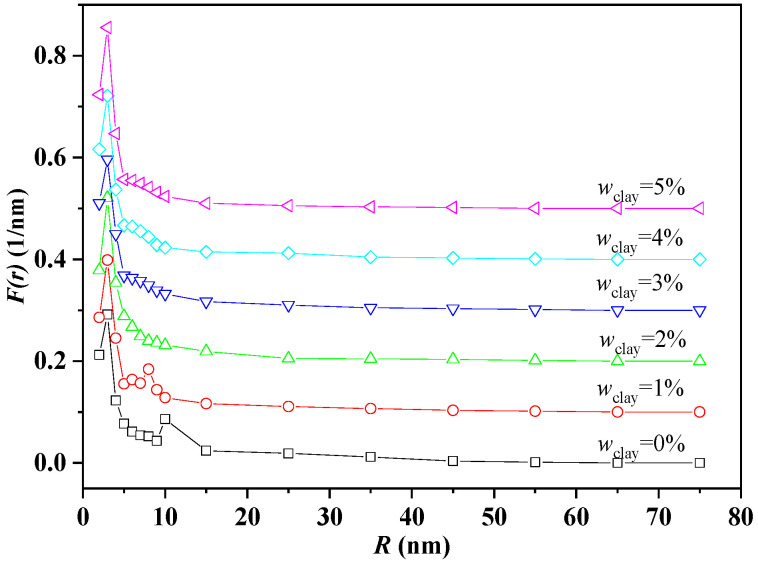
Pore size distribution of PVDF/clay nanocomposite membranes with various *w*_Clay_.

Figure 10 is the pore size distribution (PSD) of PVDF/clay nanocomposite membranes with various clay contents. The PSD results indicate that PC0 and PC1 exhibit bimodal pore size distributions, whereas PC2-PC5 show unimodal distributions. All membranes display a characteristic peak at 3 nm, with PC0 and PC1 showing additional peaks at 10 nm and 6 nm respectively. As clay content increases, the pore size distribution gradually narrows, which corresponds to the enhancement trend of the protein rejection rate. The wet membrane thickness was fixed at 200 μm during casting. After drying, the dry thickness of PVDF/OMMT membranes was slightly higher than that of neat PVDF, indicating that OMMT platelets suppress membrane shrinkage and improve dimensional stability. The increased porosity and pore connectivity are mainly attributed to the nucleation effect of OMMT, the suppressed shrinkage, and the pore-forming function of LiCl. These structural variations contribute to the improved permeability and stable rejection performance. Compared with previous studies on PSf/clay composite membranes [20], the PVDF/clay nanocomposite membranes in this work exhibit a more significant enhancement in pure water flux and a stable improvement in protein rejection. This can be attributed to the uniform exfoliation of OMMT and the optimized membrane pore structure.

### 3.6. Thermal Properties and Mechanical Properties

#### 3.6.1. Thermal Properties

**Figure 11:**
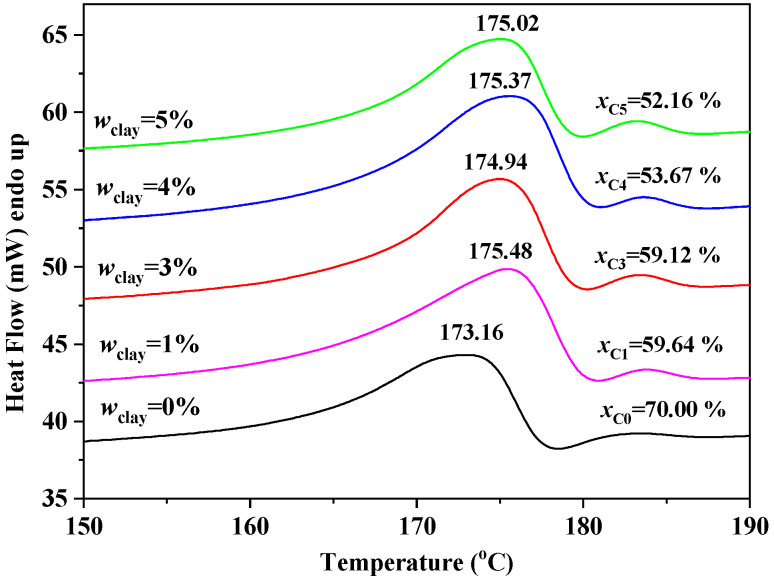
DSC thermograms of PVDF/clay nanocomposite membranes with different *w*_Clay_.

Figure 11 shows the DSC thermograms of PVDF/clay nanocomposite membranes with different clay contents. The DSC analysis results indicate that as the clay content increases from 0 to 5 wt%, the melting point (*T*_m_) of PVDF rises from 173.16 °C to 175.48 °C (an increase of approximately 2.32 °C), while the crystallinity (*X*_C_) decreases from 70.00% to 52.16%. Hydrogen bonding and physical entanglement between OMMT and PVDF chains alter the crystallization behavior of the polymer matrix. As a heterogeneous nucleating agent, OMMT remarkably raises nucleation density and favors the generation of more perfect crystallites. Nevertheless, high nucleation density restricts crystal growth and introduces abundant tie chains between adjacent crystallites, which accounts for the reduced overall crystallinity. In addition, the melting temperature of all tested membranes exhibits slight variations and is considered equivalent within experimental error. No additional crystal transformation or structural change is observed.

**Figure 12:**
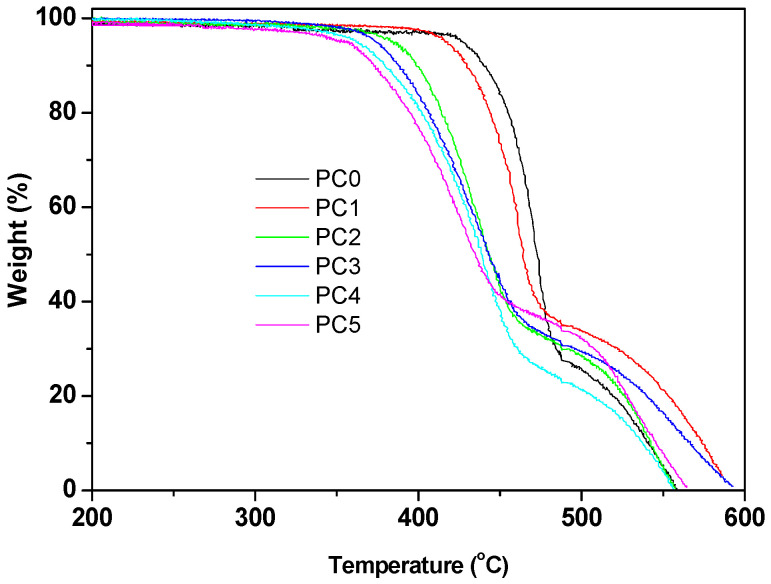
TGA curves of PVDF/clay nanocomposite membranes with various *w*_Clay_.

Figure 12 shows the TGA curves of PVDF/clay nanocomposite membranes with various clay contents. The TGA results indicate that the thermal degradation process of all membranes occurs in two stages, with the major weight loss interval ranging from 350 to 600 °C, corresponding to the decomposition of polymer backbones. As clay content increases, the temperature for 5 wt% weight loss decreases gradually from 426 °C for PC0 to 354 °C for PC5, indicating that clay incorporation reduces the thermal stability of the membranes. This is attributed to metal oxides such as Al_2_O_3_ and CaO present in clay reacting with HF, a decomposition product of PVDF, thereby promoting further degradation of PVDF molecules [18]. Despite this reduction in thermal stability, the temperature for 5 wt% weight loss remains above 350 °C, which satisfies the operational temperature requirements for practical water treatment applications.

**Figure 13:**
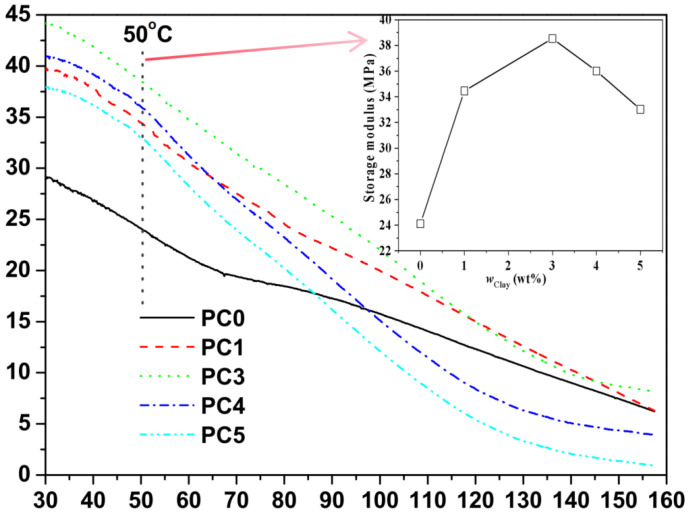
Temperature dependence of the storage modulus for PVDF/clay nanocomposite membranes.

Figure 13 presents the temperature dependence of the storage modulus for PVDF/clay nanocomposite membranes with different clay contents. DMA results (50 °C) show that the storage modulus of the PC3 membrane (3 wt% clay) is 38.54 MPa, which is 59.85% higher than that of the neat PVDF membrane (24.11 MPa). When clay content exceeds 3 wt%, the storage modulus decreases to 33.02 MPa (PC5), which is still 37.0% higher than that of the neat PVDF membrane, but lower than that of PC3. This is because excessive clay leads to over-increased porosity (53.1% for PC5 vs. 48.7% for PC3), weakening the membrane matrix structure. The exfoliated dispersion of clay layers enhances the structural strength of the membranes, while variations in storage modulus reflect changes in membrane porosity. When the clay content exceeds 3 wt%, a significant porosity increase leads to structural weakening of the membrane matrix, counteracting the reinforcing effect of clay platelets and consequently causing a decline in storage modulus.

#### 3.6.2. Tensile Strength and Elongation at Break

**Figure 14:**
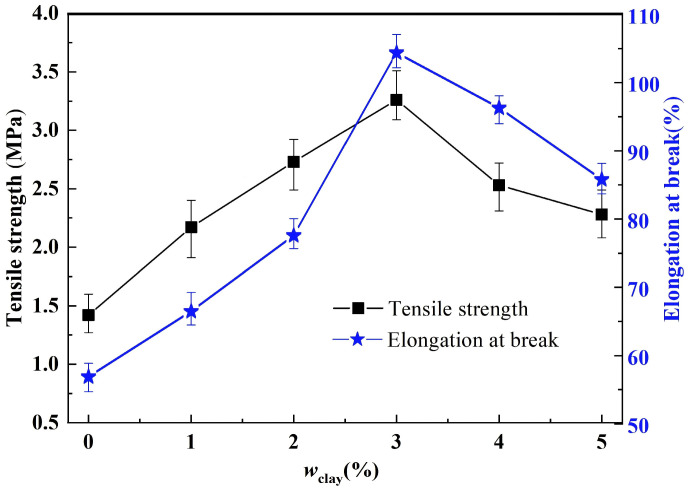
Tensile strength and elongation at break of PVDF/clay nanocomposite membranes as functions of OMMT loading.

The tensile strength and elongation at break of PVDF/clay nanocomposite membranes with different OMMT loadings are shown in Figure 14.

With increasing OMMT content from 0 to 3 wt%, the tensile strength gradually increases and reaches the maximum value at 3 wt%, indicating that appropriate OMMT loading effectively enhances the mechanical strength of the membrane. The uniformly exfoliated OMMT platelets form strong interactions with PVDF molecular chains, which transfer stress and restrict crack propagation, thereby improving tensile properties.

However, when the OMMT content exceeds 3 wt%, the tensile strength begins to decrease slightly. This is attributed to the excessive increase in membrane porosity and the slight agglomeration of clay particles, which weaken the structural integrity of the polymer matrix.

The elongation at break shows a similar trend: it increases with OMMT loading below 3 wt% and decreases at higher loadings. The moderate increase in elongation at break suggests that the addition of a suitable amount of OMMT improves the toughness of the membrane, while excessive clay causes rigidity and brittleness.

These results confirm that 3 wt% is the optimal OMMT loading, at which the PVDF/clay nanocomposite membrane achieves the best balance between tensile strength, elongation at break, and storage modulus.

### 3.7. Antifouling Study

**Figure 15:**
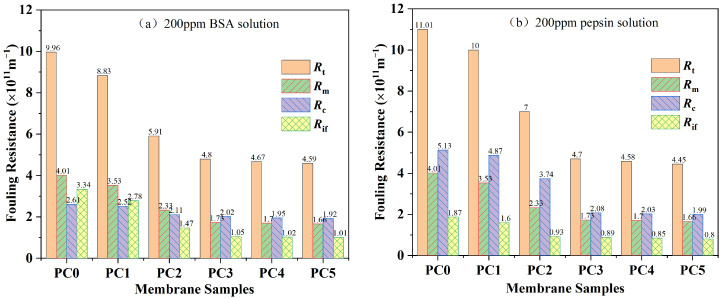
Different filtration resistance of PVDF/clay nanocomposite membranes (**a**) BSA solution, and (**b**) pepsin solution.

Figure 15 presents the different filtration resistances of PVDF/clay nanocomposite membranes with different clay contents in the filtration of BSA solution and pepsin solution. The antifouling experimental results (Figure 15 and Figure S2) demonstrate that with increasing clay content, the permeation flux of the nanocomposite membrane during BSA and pepsin solution filtration significantly improves, accompanied by reduced flux decline after fouling and markedly enhanced flux recovery rate following hydraulic cleaning. Resistance analysis reveals that total resistance (*R*_t_), intrinsic membrane resistance (*R*_m_), cake layer resistance (*R*_c_), and irreversible fouling resistance (*R*_if_) all decrease with higher clay content. The improved antifouling performance can be attributed to three factors: (1) Higher hydrophilicity reduces protein adsorption; (2) Increased surface roughness and surface area enhance water permeability and weaken foulant adhesion; (3) More negative surface charge strengthens electrostatic repulsion against negatively charged proteins, mitigating pore blocking and irreversible fouling.

Due to the molecular size of pepsin (approximately 2.8 nm) being smaller than that of BSA (approximately 4.0 nm) and closer to the average membrane pore size (3 nm), pepsin molecules tend to form a dense cake layer on the membrane surface during filtration, resulting in higher *R*_c_ compared to BSA filtration. However, pepsin exhibits a weaker adsorption capacity on the membrane surface, leading to lower *R*_if_ than that observed in BSA filtration. The enhanced antifouling performance of the nanocomposite membrane was further confirmed through calculations of flux recovery rate (*F*_r_) and permeate flux decline rate. The BSA flux recovery rate of the PC3 membrane reached 89.2%, representing a 36.6% improvement compared to the PC0 membrane (65.3%); the permeate flux decline rate decreased from 42.1% for PC0 to 18.7% for PC3.

### 3.8. Long-Term Stability and Environmental Safety

The long-term stability test results for PC0 and PC3 membranes (Figure S3) showed that the pure water flux of the PC3 membrane decreased from 208.6 L·m^−2^·h^−1^ to 162.3 L·m^−2^·h^−1^ during continuous operation over 30 days, with a flux decline rate of 22.2%, which is lower than that of the PC0 membrane (29.3%), indicating good long-term operational stability of the membrane. Environmental safety test results for the PC3 membrane (Table S1, Supplementary File S2) demonstrated that the dissolution concentrations of Al and Ca in the membrane were 0.02–0.04 mg/L and 0.01–0.02 mg/L, respectively, during the 30-day operation period, which were far below the health standard limits for drinking water.

### 3.9. Economic Analysis

The preparation cost analysis of PVDF/clay nanocomposite membranes reveals that the raw material cost of the nanocomposite membrane with 3 wt% clay content increases by approximately 0.51% compared to neat PVDF membranes (Table S2, Supplementary File S3). However, due to enhanced flux and improved antifouling properties, the nanocomposite membrane enables a reduction in both membrane module quantity and cleaning frequency during practical applications, thereby lowering operational costs. It is estimated that water treatment systems employing PVDF/clay nanocomposite membranes achieve comprehensive total investment and operational cost savings of approximately 28.1% and 5.4% relative to neat PVDF membrane systems, exhibiting good economic feasibility (Tables S3 and S4, Supplementary File S3). Furthermore, as a naturally abundant mineral resource, clay offers wide availability and low cost. The existing membrane production processes can be directly applied for large-scale manufacturing of PVDF/clay nanocomposite membranes without requiring significant modifications, indicating promising industrial application prospects.

### 3.10. Comparison with Previous Research

Table 4 is a comparison of key findings between this study and previous PVDF/clay membrane research. Table 4 presents a comparative analysis of this study with previous PVDF/clay nanocomposite membrane research, using the NIPS method as the preparation method. The comparison covers three aspects: clay types and addition amounts, core performance findings, and research limitations. It clearly outlines the progress and common shortcomings in this field, while highlighting the innovative breakthroughs and technical advantages of this study. This provides a practical reference for the development, process optimization, and practical application of PVDF/clay ultrafiltration membranes.

Previous research on PVDF/clay membranes has encountered several common challenges. Firstly, the selection of clay materials predominantly relies on OMMT, with a small proportion of dopamine-modified sodium-based montmorillonite (D-MMT). However, high addition levels often lead to agglomeration, which remains a widespread bottleneck. Different modified clays exhibit low agglomeration thresholds, with conventional OMMT showing performance degradation beyond 4 wt%, and even studies achieving 5 wt% addition demonstrate poor long-term stability. Secondly, performance studies tend to be singular, focusing narrowly on single-dimensional improvements while lacking comprehensive characterization of membrane morphology, crystal form, separation efficiency, and anti-contamination properties. Additionally, the quantitative-effect relationship between clay content and performance is often overlooked. Thirdly, membrane performance exhibits significant limitations, frequently presenting trade-offs between flux and rejection, mechanical strength and formability. Some modified membranes exhibit pure water flux far below practical application requirements. Fourthly, insufficient validation for practical applications remains prevalent, with research limited to laboratory performance characterization. Long-term operation, environmental safety, and economic analyses are lacking, and the feasibility of large-scale production has not been explored. Lastly, crystalline form regulation and structural-performance mechanisms remain underexplored. Only a few studies have investigated the clay-induced effects on PVDF crystal form, failing to elucidate the correlation between clay content and PVDF crystal form retention and lacking systematic theoretical explanations.

This study employs OMMT as the modified clay with an addition range of 0–5 wt%, achieving multidimensional breakthroughs in this field without significant research limitations. Firstly, the optimized casting solution preparation process enables uniform dispersion of OMMT in the delaminated state across the full 0–5 wt% range, overcoming the technical bottleneck of high addition amount agglomeration. Secondly, it systematically investigates the impact of clay content on membrane multidimensional properties for the first time, clarifying the evolution patterns of each performance parameter and filling the gap in quantity-effect relationship analysis. Thirdly, it reveals the regulatory mechanism of delaminated clay as a heterogeneous nucleation site maintaining the dominant crystalline structure of β form PVDF, addressing the shortcomings in crystalline structure research. Fourthly, the optimal addition amount of 3 wt% is determined, achieving synergistic improvements in membrane flux, protein retention, mechanical strength, and anti-contamination performance, with thermal stability meeting practical water treatment requirements. Fifthly, comprehensive validation of long-term stability, environmental safety, and economic viability is completed. The 3 wt% nanocomposite membrane material cost increases by only 0.51%, significantly reducing overall costs while the preparation process is compatible with existing industrial production lines, demonstrating potential for large-scale application.

### 3.11. Practical Application

The PVDF/clay nanocomposite membrane developed in this work breaks through the bottleneck of poor hydrophilicity and serious fouling of traditional PVDF membranes, and its performance optimization is closely combined with the actual demand of water treatment engineering. The integration of this membrane material into real-world applications is not only reflected in the direct replacement and upgrading of existing membrane units but also in the adaptation to different water treatment scenarios such as wastewater purification with high protein content and compatibility with industrial production systems. At the same time, the environmental safety and economic feasibility verified by the study provide a solid engineering basis for its large-scale promotion and application. In the context of the global water resource shortage and the increasing demand for water treatment efficiency and environmental protection, this PVDF-based nanocomposite membrane has important application value for promoting the technological upgrading of the water treatment industry and realizing the sustainable development of water resource utilization.

## 4. Conclusions

In this work, flat-sheet PVDF nanocomposite ultrafiltration membranes containing 0–5 wt% OMMT were successfully fabricated via the NIPS method. OMMT was uniformly exfoliated and dispersed within the PVDF matrix, and all nanocomposite membranes maintained a dominant β crystalline modification without crystal transformation. The incorporation of OMMT effectively regulated membrane morphology, leading to increased porosity, improved pore connectivity, and enhanced surface hydrophilicity. With increasing OMMT content, the pure water flux and protein rejection of the membranes were significantly improved. At 5 wt% OMMT loading, the pure water flux reached 216.3 L·m^−2^·h^−1^, and the rejection rates of BSA and pepsin reached 98.6% and 95.1%, respectively.

The optimal OMMT loading was determined to be 3 wt%, at which the membrane exhibited the highest storage modulus (increased by 59.8%), optimal tensile strength, and favorable elongation at break. The nanocomposite membranes showed remarkably reduced filtration resistance, enhanced flux recovery rate, and improved antifouling properties due to the increased hydrophilicity, optimized pore structure, and more negative surface charge. Long-term operation tests confirmed the superior stability of the 3 wt% OMMT membrane, and environmental safety assessment verified that ion leaching was far below the standard limits for drinking water. Economic analysis indicated that the nanocomposite membranes only slightly increased material costs but greatly reduced the overall investment and operating expenses in practical water treatment systems.

This study demonstrates that OMMT is a high-efficiency, low-cost, and eco-friendly modifier for PVDF membranes. The resulting nanocomposite membranes exhibit balanced permeability, selectivity, mechanical strength, antifouling performance, long-term stability, and economic feasibility, showing great potential for sustainable water treatment applications.

## Figures and Tables

**Table 1:** Composition of the casting solution.

Membranes	PVDF (g)	DMAc (g)	Clay (g)	LiCl (g)	*w*_clay_ * (wt%)
PC 0	15.00	60.00	0	3.00	0
PC 1	15.00	60.00	0.15	3.00	1
PC 2	15.00	60.00	0.30	3.00	2
PC 3	15.00	60.00	0.45	3.00	3
PC 4	15.00	60.00	0.60	3.00	4
PC 5	15.00	60.00	0.75	3.00	5

* *w*_clay_ is the mass ratio of clay to PVDF.

**Table 2:** Surface area difference and roughness parameters of PVDF membranes with various OMMT loadings.

*w*_clay_/%	ds/%	*R*_q_/nm	*R*_a_/nm	*R*_tm_/nm
0	4.29	7.51	5.94	53.8
3	8.55	11.5	9.35	66.4

**Table 3:** Zeta potential of PVDF/OMMT nanocomposite membranes at pH 7.0 ± 0.1.

*w*_clay_/%	Zeta Potential/mV
0	−16.9 ± 0.2
3	−25.3 ± 0.3

**Table 4:** Comparison of key findings between this study and previous PVDF/clay membrane research via NIPS method.

Study	Clay Type and Loading	Key Findings	Limitations
Li & Kim [18]	Modified MMT, varied	Improved thermal degradation resistance; suitable for prolonged high-temperature service.	No data on membrane transport or fouling properties.
Hwang et al. [19]	Nanoclay, varied	Enhanced mechanical strength in porous PVDF membrane supports.	Limited focus on separation performance.
Rajabi et al. [20]	OMMT, varied	Improved hydrophilicity, flux, antifouling; intercalated structure; higher porosity.	No systematic study on clay content effects.
Farahani et al. [35]	Cloisite 30B (OMMT), varied	0.5 wt% OMMT loading increases the water flux of PVDF membranes by 187%, with significant improvements in hydrophilicity and porosity. High loading (>4 wt%) leads to nanoparticle aggregation, resulting in decreased flux, and nanoparticle embedding reduces the membrane elongation at break.	High nanoparticle loading (>4 wt%) promotes agglomeration, impairing separation performance; Nanoparticle embedding reduces membrane elongation at break and weakens mechanical flexibility.
Navarro-Tovar et al. [36]	Clay Type: Cloisite 20A (OMMT), varied	The membrane exhibits optimal performance at a 4 wt% OMMT loading: porosity ≈ 93%, contact angle ≈ 147° (superhydrophobic), and mechanical strength (55 MPa) surpasses commercial membranes, making it suitable for membrane distillation desalination.	High OMMT loading increases the viscosity of the spinning solution, which may affect fiber uniformity.
Rezaei- DashtArzhandi et al. [37]	Hydrophobic montmorillonite (OMMT), varied	OMMT thins the skin layer, elongates the finger-like pores, enhances N_2_ permeability, and increases CO_2_ absorption flux, with optimal long-term stability at a 5 wt% loading. The contact angle increases with rising OMMT loading, while PVDF membranes exhibit excellent mechanical stability. These properties make them suitable for membrane distillation desalination.	OMMT tends to aggregate under high load and exhibits poor long-term stability.
Lai et al. [38]	Cloisite15A (OMMT)	The nano-clay induced the transformation of PVDF crystal form from α form to β form, which improved the stiffness and toughness of the membrane, thereby enhancing its wear resistance. Low-concentration nano-clay reduced the pure water flux of the membrane, while high loading (5 wt%) slightly increased the flux but exacerbated clay agglomeration, significantly decreasing the ductility and toughness of the membrane.	Nano-clay loading exceeding 1 wt% will lead to agglomeration, which reduces ductility and toughness of the membrane, while its wear resistance decreases. Without the addition of pore-forming agents, the water flux of the membrane is significantly lower than that of conventional commercial membranes, failing to directly meet practical application requirements.
Lai et al. [39]	8 Commercial Modified Montmorillonite	Nanomer I.30E clay exhibited the highest retention rate in the membrane (43%), with its mono-alkyl chain-containing organic modifier demonstrating optimal compatibility with the PVDF matrix. Hydrophilic modified clays (Cloisite10A/30B, NanomerI.34TCN) readily induced the formation of a high proportion of β-form in PVDF, but promoted the formation of large voids in the membrane, resulting in a looser membrane structure and reduced mechanical properties.	Severe loss of nano-clay during membrane casting not only increases production costs but also poses environmental risks.
Cai et al. [40]	Dopamine-modified sodium montmorillonite (D-MMT)	Dopamine modification effectively inhibits clay agglomeration, with D-MMT being uniformly dispersed in the PVDF matrix. Appropriate D-MMT loading can increase the tensile strength, the elongation at break and the water flux. The hydrophilicity of D-MMT promotes phase separation, transforming the membrane from a large void structure of neat PVDF to a sponge-like structure, thereby enhancing its mechanical properties and hydrophilicity.	D-MMT with excessive loading (>0.8 wt%) tends to aggregate, resulting in membrane performance degradation. There is a trade-off between flux and retention rate. The water flux is very low (15 L·m^−2^·h^−1^~22 L·m^−2^·h^−1^).
Rahmati et al. [41]	Cloisite30B (OMMT), Hydrophilic clay	The synergistic effect between clay and MWCNTs effectively inhibits the aggregation of both components in the PVDF matrix, thereby enhancing the membrane’s porosity, surface roughness, and hydrophilicity. Nanoclay demonstrates superior performance in improving the mechanical properties of the membrane compared to MWCNTs, with the hybrid membrane exhibiting significantly higher Young’s modulus and tensile strength than neat PVDF membranes.	The long-term operation stability and cleaning regeneration performance of the membrane were not investigated, and the basic data for industrial applications were lacking.
This work	OMMT, 0–5 wt%	Optimal clay content (3 wt%) enhances flux, rejection, mechanical strength, and antifouling; Systematically study the influence of clay content, clarify the mechanism of β-form retention, and balance economic and environmental benefits.	——

## Data Availability

All data generated or analyzed during this study are included in this published article.

## Supplementary Materials

The following supporting information can be downloaded at: https://www.mdpi.com/article/10.3390/polym18121424/s1, Figure S1: Schematic diagram of UF cross flow filtration experimental setup [21]; Figure S2: Fouling and cleaning experiments of PVDF/clay nanocomposite membranes (a) BSA solution; and (b) pepsin solution; Figure S3: Long-term stability test results of PC0 and PC3 membranes; Table S1: Water quality of the simulated water sample and filtrate sample in the experiment; Table S2: Cost analysis for PC0 membrane and PC3 nanocomposite membrane; Table S3: Investment analysis for PC0 membrane and PC3 membrane using in a water treatment plant; Table S4: Operating cost for PC0 membrane and PC3 membrane using in a water treatment plant. The references [22,42,43,44,45,46,47] are cited in Supplementary Materials.

## Nomenclature and Units

*A*	the membrane filtration area (m^2^)
*A*_d_	the area of membrane in wet state (cm^2^)
*C*_f_	the concentrations of protein in initial feed (mg/L)
*C*_p_	the concentrations of protein in permeate (mg/L)
*f*(*r*)	the pore size distribution function (m^−1^)
*F*_r_	the flux recovery (%)
Δ*H*_m_	the heat of fusion for hybrid membranes with different amounts of nanoclay (J/g)
Δ*H*_m,p_	the heat of fusion of a perfect crystal taken as 104.7 J/g for neat α–PVDF (J/g)
*L*	the thickness of the membrane (m)
*M*_n_	Number-average molecular weight
*M*_w_	Weight-average molecular weight
*m*_d_	the dry sample mass (g)
*m*_w_	the wet sample mass (g)
*N*_T_	the total number of pores (dimensionless)
*P*	porosity (%)
Δ*P*	the pressure drop across the membrane (N/m^2^)
*Q*	volume flowrate of testing fluid through membrane (m^3^/s)
PF	permeation flux (L·m^−2^·h^−1^)
PWF	pure water flux (L·m^−2^·h^−1^)
*R*	rejection (%)
*R*_a_	the arithmetic mean roughness (nm)
*R*_q_	the root mean square roughness (nm)
*R*_t_	the total resistance (m^−1^)
*R*_tm_	the maximum profile height (nm)
*R*_m_	the membrane resistance (m^−1^)
*R*_if_	the irreverible fouling resistance (m^−1^)
*R*_c_	the cake resistance (m^−1^)
*r*	the radius of the wetting membrane (m)
*t*	the filtration time (h)
*T*_m_	melting temperature (°C)
*TMP*	the trans membrane pressure (Pa)
*V*	the permeate volume (L)
*w*	the mass fraction of PVDF (%)
*w*_clay_	the mass ratio of clay to PVDF (%)
*X*_C_	the degree of crystallization (%)
*ρ*_w_	the density of pure water (g/cm^3^)
*δ*	the thickness of membrane in wet state (cm)
*σ*	the surface tension of the liquid–liquid displacement mixture at 20 °C (mN/m)
*μ*	the viscosity of the displacing liquid (Pa·s)
*μ*_p_	the viscosity of permeate (Pa·s)
*τ*	the tortuosity of the pores
*θ*	the contact angle (Degree)
