# Supplementary material for: Effect of Organoclay Content on the Physicochemical and Separation Properties of PVDF/Clay Nanocomposite Membranes
Source: Polymers (Basel). 2026 Jun 7;18(12):1424. doi: 10.3390/polym18121424 (PMC13306339; doi:10.3390/polym18121424)
Supplement: Supplementary file 1 [file polymers-18-01424-s001.zip › polymers-4346938-supplementary.pdf]

Effect of organoclay content on the physicochemical and separation properties of PVDF/clay nanocomposite membranes

Jun Zhang[1,†], Boming Fan[1,†], Fengmei Shi[2,†], Chao Lin[3,†], Shuqi Ma[1], Qi Shen[1], Jinglong Yuan[1], Hua Fan[4], Yuxin Ma[1,*]

[1] College of Civil Engineering, Heilongjiang University, Harbin 150080, PR China；
[2] Heilongjiang Academy of Black Soil Conservation and Utilization, Heilongjiang Academy of Agricultural Sciences, Harbin 150086, PR China;
[3] College of Airspace Engineering, Harbin Engineering University, Harbin 150001, PR China；
[4] Research and Development Centre, Shandong Aisen Water Industry Co., Ltd., Taian 271021, PR China.
† These authors contributed equally to this work.
* Correspondence: 2010055@hlju.edu.cn; oucmyx@126.com

Academic Editor: Paola Bernardo

Received: 14 May 2026
Revised: 31 May 2026
Accepted: 4 June 2026
Published: 7 June 2026

Copyright: © 2026 by the authors. Submitted for possible open access publication under the terms and conditions of the Creative Commons Attribution (CC BY) license.

Supplementary file S1

Characterization of membranes

(1) Morphologies study

The cross-sectional morphologies of the membranes were examined using a HITACHI S–3400N SEM with an acceleration voltage of 5.0kV. The upper surface SEM images of PVDF/clay nanocomposite membranes were observed using a JEOL JEM-6700F SEM with an acceleration voltage of 20.00kV. To prepare for observation, the membranes were fractured in liquid nitrogen and subsequently coated with a thin gold layer via sputtering.

The membranes to be examined by TEM were embedded into epoxy resin capsules. The epoxy resin was microtomed with a Leyca Ultracut–R into 60–90–nm–thick slices in liquid nitrogen and then the slices were observed with a JEOL–JEM–2010 TEM, which has an acceleration voltage of 200 kV.

(2) Crystallity study

Fourier-transform infrared spectroscopy (FTIR) analysis was performed on a Perkin Elmer Spectrum 2000 spectrometer with a resolution of 1cm^{-1}. Infrared spectra were obtained for neat PVDF and PVDF/clay nanocomposite membranes from 4000 to 600 cm^{-1} using attenuated total internal reflection.

A wide–angle X–ray diffraction (XRD) study of the membranes was conducted with a Dmax–rB X–ray diffractometer under a voltage of 40 kV and a current of 30 mA using Cu K_{α} radiation (λ=0.154 nm). All samples were analyzed in a continuous scan mode with the 2 theta from 2 to 60°.

Differential scanning calorimetry (DSC) was performed to study the melting and crystallization behavior of the hybrid membranes with a Perkin Elmer DSC–7C. The temperature and energy scales were calibrated with the standard procedures. The sample was heated to 200 °C at a rate of 10 °C/min in nitrogen atmosphere and held there for 10 minutes. Then, it was cooled to 25 °C in nitrogen environment at the same rate. The melting and crystallization parameters were determined from the heating and cooling scans, respectively.

(3) Porosity (*P*) and Contact angle (*CA*)

Membrane porosity was measured in the method of dry–wet weight. The membrane preserved in distilled water was weighed after mopping superficial water with filter paper. Then the wet membrane was placed in an air–circulating oven at 60°C for 24 h and then further dried in a vacuum oven at 80°C for 24 h before measuring the dry mass. From the two masses (the wet sample mass and the dry sample mass), the porosity of membrane was calculated using formula (1) as

$$P(\%)=\frac{m_{\mathrm{w}}-m_{\mathrm{d}}}{\rho_{\mathrm{W}}\times A_{\mathrm{d}}\times\delta}\times 100 \qquad (1)$$

where P is the porosity of membrane, m_{w} is the wet sample mass (g), m_{d} is the dry sample mass (g), ρ_w is the density of

pure water (g/cm^3), A_d is the area of membrane in the wet state (cm^2) and δ is the thickness of membrane in the wet state (cm). In order to minimize experimental error, each membrane was measured for three times and calculated the average.

The contact angle measurements were carried out with a contact angle meter (DSA100, KRÜSS). A water droplet was placed onto a flat homogeneous membrane surface and the contact angle of the droplet with the surface was measured. The reported values were the averages of the contact angles of five droplets.

(4) Permeation flux (*PWF*) and rejection (*R*) and pore size distribution (*PSD*)

The permeation flux and rejection of the prepared membranes were measured by an UF cross flow filtration experimental set–up fed with deionized water at a transmembrane pressure of 100 kPa after pre–pressurized for 30 min at 200kPa. The schematic of UF cross flow filtration experimental set–up was presented in Fig. S1[1]. The permeation flux was defined as formula (2) .

$$PWF = \frac{V}{A \times t} \qquad (2)$$

where *PWF* is the pure water flux (L·m^{-2}·h^{-1}), *V* is the permeate volume (L), *A* is the membrane filtration area (m^2) and *t* is the filtration time (h).

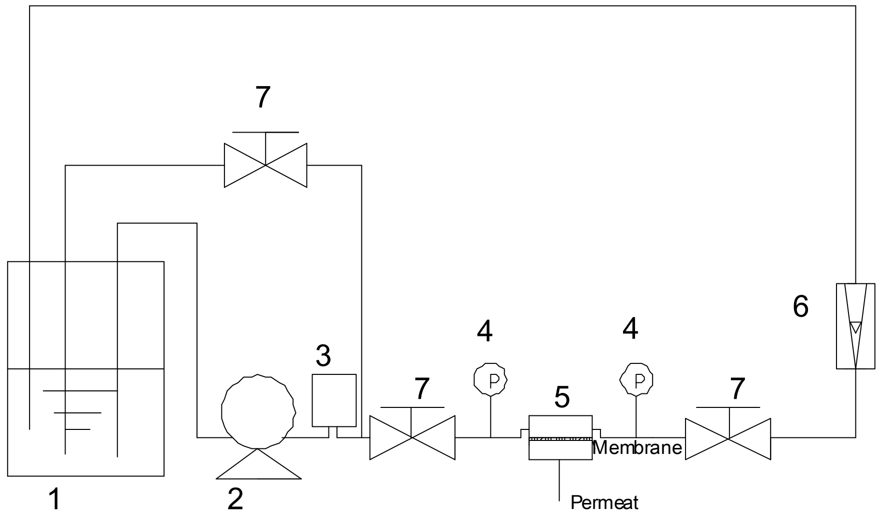

1. Feed tank 2. Booster pump 3. Buffering tank 4. Pressure gauge 5. Flat membrane cell 6. Flowmeter 7. Valve

Figure S1 Schematic diagram of UF cross flow filtration experimental setup [1]

Rejection was characterized with 200 mg/L BSA aqueous solution and 200 mg/L pepsin aqueous solution respectively after the membrane was previously filtered with the deionized water until flux was steady. The concentrations of BSA and pepsin in permeate and feed were determined by an UV–spectrophotometer (Shimadzu UV–2450, Japan). It was calculated according to formula (3).

$$R = 1 - \frac{C_p}{C_f} \qquad (3)$$

where C_p and C_f are the concentrations of protein in permeate and initial feed, respectively.

Liquid-liquid displacement method was adopted to determine the pore size distribution of different membranes. The liquid displacement method was known as combined bubble pressure and solvent permeability method. In this method, the membrane was wetted previously with an appropriate penetrating liquid and then an immiscible liquid that did not wet the membrane was pressurized to pass through the pores displacing the previous liquid which was already occupying the pores. In this work, the alcohol–rich phase of water–isobutanol–methanol (25:15:7, v/v) mixture was taken as a wetting liquid, and aqueous–rich phase of the mixture was used as a displacing liquid. The contact angle between the liquid–liquid interface and membrane material can be assumed as zero, the pore size was calculated by Cantor's equation as follows [2].

$$r = \frac{2\sigma}{\Delta P} \qquad (4)$$

where r is the radius of the wetting membrane (m), σ is the surface tension of the liquid–liquid displacement mixture at 20 °C (mN/m), ΔP is the pressure drop across the membrane (N/m^2).

The flowrate Q through a pore with the radius of r under a pressure of ΔP was calculated by Hagen–Poiseuille equation.

$$Q = \frac{\pi \times r^4 \times \Delta p}{8\mu \times L \times \tau} \qquad (5)$$

Here μ is the viscosity of the displacing liquid, L is the thickness of the membrane (m) and τ is the tortuosity of the pores.

The pore size distribution function f(r) was given by McGuire et al. [3]:

$$f(r) = \left(\frac{dQ}{d(\Delta p)} - \frac{Q}{\Delta p}\right)\frac{\Delta p^5}{C_1^5 C_2} \qquad (6)$$

Here C_1=2σcosθ, θ is the contact angle, $C_2 = \frac{N_T \pi}{8\mu L \tau}$ and N_T was the total number of pores. In this study, surface tension of the system was 0.35 mN/m at 20 °C, and τ was 1 [4].

(5) Thermogravimetric analysis (TGA) and Dynamic mechanical analysis (DMA)

Thermogravimetric analysis (TGA) was performed under nitrogen flows from 30 to 600 °C at a heating rate of 20 °C/min by using a Perkin-Elmer TGA 7.

Dynamic mechanical analysis (DMA) was performed on the membrane samples using a DMA Q 800 analyzer from TA instruments under tension film mode in the temperature of 20 to 150 °C at a fixed frequency of 1 Hz and heating rate of 3 °C/min.

(6) Fouling analysis

Membrane fouling mainly resulted from the protein adsorption onto the membrane surface. Protein fouling of the membrane was a complicated process because of the complex interaction between the protein and the membrane surface. BSA is one of the most widely studied proteins. The albumin has the ability to bind reversibly to the variety of ligands. Therefore many studies have used BSA as model protein for systematically studying the fouling behavior of UF/MF membranes [5]. The batch filtration experiment was performed using 200ppm BSA solution to achieve the fouling of the membranes.

The procedure of fouling experiment was similar to that described by Kang et al. [6]. In the fouling experiment, the cross-flow filtration experiment setup was used. The experiments were carried out under the same transmembrane pressure (*TMP*=100 kPa). Each membrane was compacted with deionized water until the permeate flux became constant. The pure water flux was recorded. Then, the reservoir was emptied and the contaminant solution was poured. The time-varying permeate flux (*PF*) was measured. After 180 min, the membrane was rinsed with deionized water and the flux recovery was measured. The degree of membrane fouling was quantitatively calculated using the resistance in series model as given below [7,8].

$$R_t = \frac{TMP}{\mu_{\mathrm{p}} J_{\mathrm{fw}}} = R_{\mathrm{m}} + R_{\mathrm{c}} + R_{\mathrm{if}} \qquad (7)$$

where J_{fw} is the steady state permeation flux ($\mathrm{m^3/m^2 \cdot s}$) and *TMP* is the transmembrane pressure (Pa), μ_{p} is the viscosity of permeate (Pa·s) measured in distilled water after the fouling of the membrane and R_{t} is the total filtration resistance ($\mathrm{m^{-1}}$). It is assumed that total filtration resistance is the sum of intrinsic membrane resistance (R_{m}), cake resistance (R_{c}) offered by loosely bound protein (BSA) layer formed on membrane surface, irreverible fouling resistance (R_{if}) offered by foulants adsorption, strong attachment of foulants such as pore blocking, cake, gel and biofilm, and inner pore plugging. These resistances can be calculated from experimental data using following Eqs. (8)–(10):

$$R_m = \frac{TMP}{\mu_{\mathrm{p}} J_{\mathrm{w}}} \qquad (8)$$

$$R_{\mathrm{c}} = R_{\mathrm{t}} - \frac{TMP}{\mu_{\mathrm{p}} J_{\mathrm{rw}}} \qquad (9)$$

$$R_{if} = R_t - R_m - R_c \qquad (10)$$

where, J_w is the clean membrane water flux at steady state, J_{rw} is the water flux of fouled membrane measured after removing loose bound protein by rinsing membrane in water.

(7) Determination of tensile strength and elongation at break

Tensile strength and elongation at break of membranes were determined by a universal electronic strength measurement (AGS-J, Shimadzu). The membrane samples (thickness 0.2 mm, width 6 mm) were folded three times to avoid the influence of edge defects on the experimental results. Measurements were carried out at room temperature and a strain rate of 20 mm/min was employed. The reported values were the averages of at least five samples.

Supplementary file S2

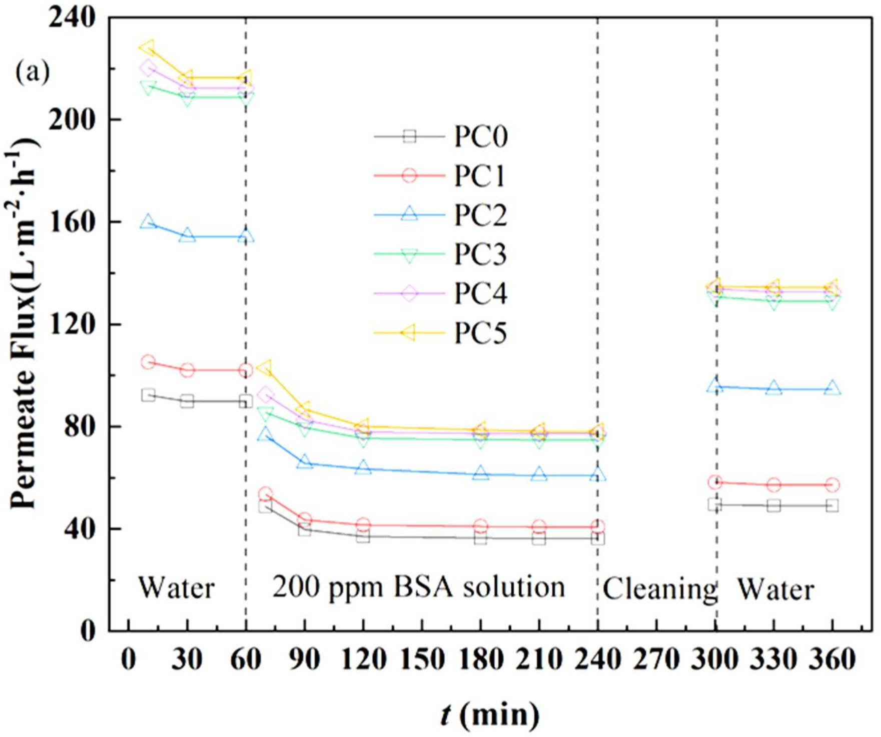

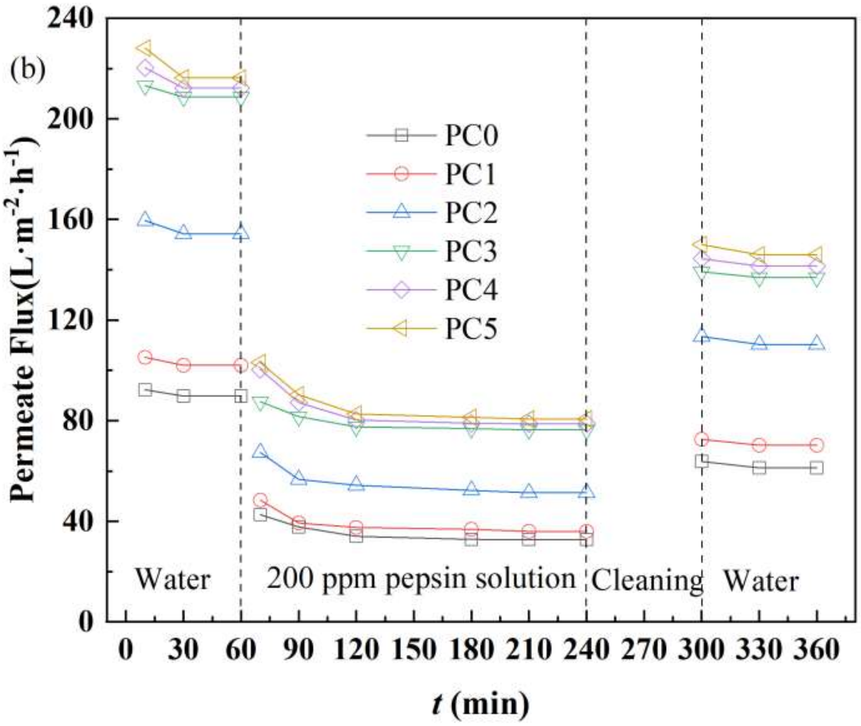

Figure S2 Fouling and cleaning experiments of PVDF/clay nanocomposite membranes (a) BSA solution; and (b) pepsin solution.

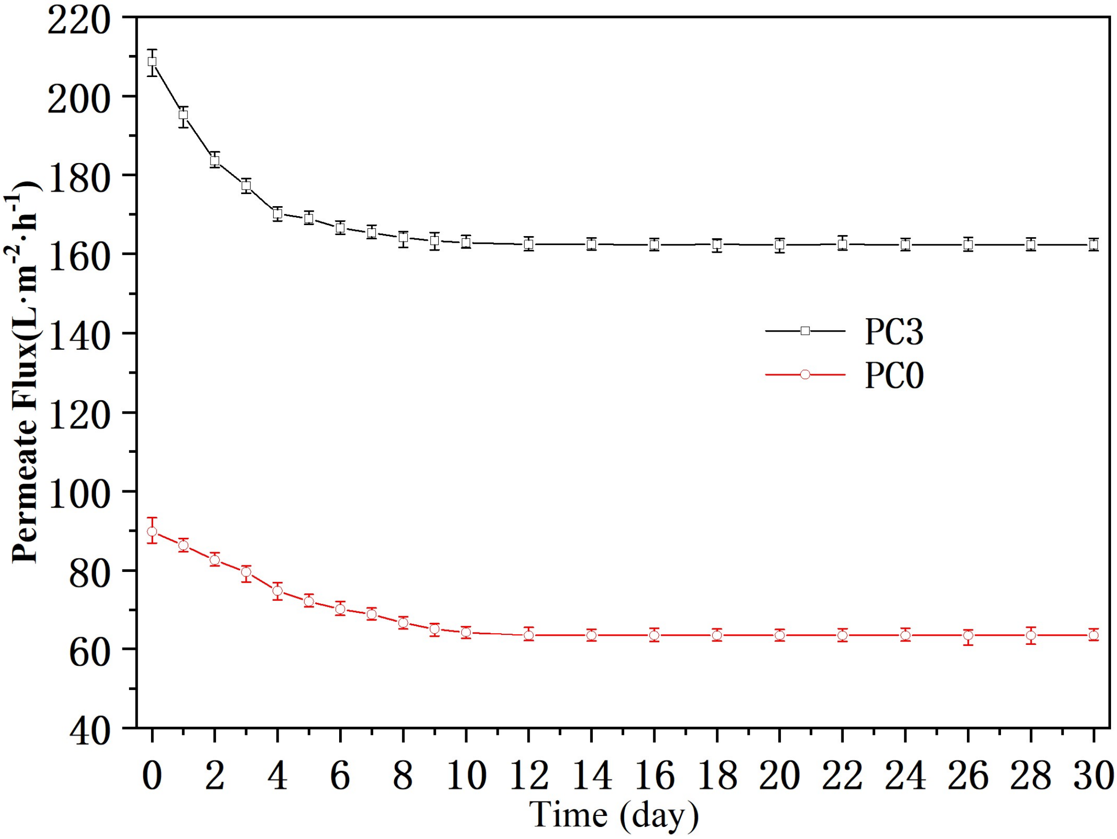

Figure S3 Long-term stability test results of PC0 and PC3 membranes

Table S1 Water quality of the simulated water sample and filtrate sample in the experiment.

Water quality	Original sample	Filtrate sample		
		10th day	20th day	30th day
Al^{3+}(mg/L)	0.012±0.002	0.015±0.003	0.015±0.002	0.016±0.002
Mg^{2+} (mg/L)	11.32±0.02	11.33±0.02	11.33±0.03	11.34±0.03
Ca^{2+} (mg/L)	14.03±0.02	14.04±0.02	14.05±0.03	14.05±0.02
Fe^{2+} (mg/L)	0.008±0.002	0.008±0.003	0.009±0.002	0.009±0.002
TOC (mg/L)	0.05±0.01	0.06±0.01	0.06±0.02	0.07±0.02
pH	7.20±0.05	7.20±0.05	7.20±0.06	7.20±0.05

Supplementary file S3

Table S2 is the cost analysis for PC0 membrane and PC3 nanocomposite membrane based on the daily production capacity of a reactor.

Table S2 Cost analysis for PC0 membrane and PC3 nanocomposite membrane

Items	Unit Price	PC0		PC3	
		Amount	Cost（¥）	amount	Cost（¥）
PVDF	120 ¥/kg	16 kg	1,920	16 kg	1,920
DMAc（solvent）	6 ¥/kg	64 kg	384	64 kg	384
DMAc（coagulation bath）	6 ¥/kg	80 kg	480	80 kg	480
LiCl	200 ¥/kg	3.2 kg	640	3.2 kg	640
Clay	50 ¥/kg	—	—	0.48 kg	24
Deioned water	4 ¥/m^3	8 m^3	32	8 m^3	32
Glycerol	1 2 ¥/kg	40 kg	480	40 kg	480
Labor expense	500 ¥/（person·d)	4 person·d	2,000	4 person·d	2,000
Electrical expense	1 ¥/kWh	2,000 kWh	3,000	2000 kWh	3,000
Module processing	200 ¥/module	7 module	1,400	7 module	1,400
Equipment depreciation and site cost	4,000 ¥/d	1d	4,000	1d	4,000
Total cost（¥）		14,336		14,410	
Membrane production（m^2）		280		280	
Unit Price（¥/m^2）		51.2		51.46（↑0.51%）	

Table S3 is the investment analysis for PC0 membrane and PC3 membrane using in a 10,000 m^3/d water treatment plant.

For a surface water treatment plant with a capacity of 10,000 m^3/d using river water as the source, the designed hourly flow rate is 417 m^3/h (based on 24-hour continuous operation and a 1.2 times fluctuation factor, resulting in an actual designed hourly flow rate of 417×1.2≈500 m^3/h). The main treatment processes are as follows:

Raw water → coarse screen → fine screen → coagulation → sedimentation→ self-cleaning filter → ultrafiltration

system (hollow fiber ultrafiltration membrane) → clear water tank → effluent

Auxiliary systems: backwash system, chemical cleaning system, dosing system, automatic control system, and aeration system (for air scrubbing)

The hollow fiber ultrafiltration membranes were fabricated using PC0 and PC3 membrane processes, with the following specifications: Design flux: 50 LMH (PC0); 80 LMH (PC3); Operation mode: Primarily dead-end filtration, with cross-flow flushing performed every 30-60 minutes to control concentration polarization and membrane fouling; Recovery rate: 90%, with concentrated water recycled to the pretreatment front end to enhance water resource utilization; Transmembrane pressure differential (TMP): Design operating TMP ranges from 0.05-0.15 MPa. Chemical cleaning is initiated when TMP increases ≥50 kPa or the standardized flux decreases ≥10%. Parameters for individual membrane modules: Water production rate: 2.0 m^3/h (PC0) and 3.2 m^3/h (PC3); Membrane area: 40 m^2 per module; Operating pressure: 0.02-0.20 MPa.

Table S3 Investment analysis for PC0 membrane and PC3 membrane using in a water treatment plant

Device	PC0		PC3	
	Specifications and Quantity	Investment (¥)	Specifications and Quantity	Investment (¥)
Coarse screening	Grid spacing 10 mm, channel width 1.2 m, 1 set	20,000	Grid spacing 10 mm, channel width 1.2 m, 1 set	20,000
Fine screening	Grid spacing 2 mm, channel width 1.0 m,1 set	30,000	Grid spacing 2 mm, channel width 1.0 m,1 set	30,000
Sedimentation tank	Tube settler，1 set	60,000	Tube settler，1 set	60,000
Self-cleaning filter	Filtration accuracy 100 μm, treatment capacity 250 m^3/h, 3 sets (2 for use, 1 standby)	90,000	Filtration accuracy 100 μm, treatment capacity 250 m^3/h, 3 sets (2 for use, 1 standby)	90,000
Hollow fiber UF membrane module (membrane area 40 m^2 per module)	Permeate flow: 2.0 m^3/h, 252 modules (including 2 spare), Sale price: 3200¥ per module	806,400	Permeate flow: 3.2 m^3/h, 160 modules (including 3 spare), Sale price: 3200¥ per module	512,000
Backwashing pump	Flow rate: 500 m^3/h, head: 15 m, 2 sets(1 for use, 1 standby)	20,000	Flow rate: 500 m^3/h, head: 15 m, 2 sets(1for use, 1 standby)	20,000
Cleaning pump	Flow rate: 200 m^3/h, head: 20 m, 2 sets (1 for use, 1 standby)	20,000	Flow rate: 200 m^3/h, head: 20 m, 2 sets (1 for use, 1 standby)	20,000
Dosing pump（PAC）	Flow rate: 50 L/h, head: 15 m, 2 sets (1 for use, 1 standby)	2,000	Flow rate: 50 L/h, head: 15 m, 2 sets (1 for use, 1 standby)	2,000
Total investment (¥)	1,048,400		754,000 (↓28.1%)	

Table S4 is the operating cost for PC0 membrane and PC3 membrane using in a water treatment plant. The operating cost is calculated based the electrical expense, chemical costs and membrane replacement costs.

Table S4 Operating cost for PC0 membrane and PC3 membrane using in a water treatment plant

Device	PC0		PC3	
	Motor power, chemical costs, material costs	Operating cost (¥/d)	Motor power, chemical costs, material costs	Operating cost (¥/d)
Coarse screening	1.5 kW*24h/d*1¥/kWh =36¥/d	36	1.5 kW*24h/d*1¥/kWh =36¥/d	36
Fine screening	1.1 kW*24h/d*1¥/kWh =26.4¥/d	26.4	1.1 kW*24h/d*1¥/kWh =26.4¥/d	26.4
Sedimentation tank	PAC cost: 0.025kg/m^3*10000m^3/d*1.5¥/kg =375¥/d	375	PAC cost: 0.025kg/m^3*10000m^3/d*1.5¥/kg =375¥/d	375
Self-cleaning filter	0.75 kW*24h/d*1¥/kWh =18¥/d	18	0.75 kW*24h/d*1¥/kWh =18¥/d	18
Hollow fiber UF membrane module	37 kW*24h/d*1¥/kWh =888¥/d Membrane replacement(5-year): 252 module*3200¥/module /(365d*5)=441.9¥/d	1,329.9	37 kW*24h/d*1¥/kWh =888¥/d Membrane replacement(5-year): 160 module*3200¥/module /(365d*5)=280.5¥/d	1,168.5
Backwash pump	37 kW*24h/d*1¥/kWh =888¥/d (Intermittent operation, calculated as 10% of the time)	888	37 kW*24h/d*1¥/kWh =888¥/d (Intermittent operation, calculated as 10% of the time)	888
Cleaning pump	18.5kW*24h/d*1¥/kWh =444¥/d (Intermittent operation, calculated as 3% of the time)	444	18.5kW*24h/d*1¥/kWh =444¥/d (Intermittent operation, calculated as 3% of the time)	444
Dosing pump （PAC）	0.75 kW*24h/d*1¥/kWh =18¥/d	18	0.75 kW*24h/d*1¥/kWh =18¥/d	18
Total cost (¥/d)	3,135.3		2,973.9	
Unit cost (¥/m^3)	0.314		0.297 (↓5.4%)	

References

1. Ma, Y.; Shi, F.; Ma, J.; Wu, M.; Zhang, J.; Gao, C. Effect of PEG additive on the morphology and performance of polysulfone ultrafiltration membranes. Desalination 2011, 272, 51–58. https://doi.org/10.1016/j.desal.2010.12.054.
2. Calvo, J.I.; Bottino, A.; Capannelli, G.; Hernández A. Comparison of liquid–liquid displacement porosimetry and scanning electron microscopy image analysis to characterize ultrafiltration track–etched membranes, J. Membr. Sci. 239 (2004) 189–197. https://doi.org/10.1016/j.memsci.2004.02.038.
3. McGuire, K.S.; Lawson, K.W.; Lloyd, D.R. Pore size distribution determination from liquid permeation through microporous membranes, J. Membr. Sci. 99 (1995) 127–137. https://doi.org/10.1016/0376-7388(94)00209-H.
4. Piątkiewicz, W.; Rosińskia, S.; Lewińska, D.; Bukowski, J.; Judycki, W. Determination of pore size distribution in hollow fibre membranes, J. Membr. Sci. 153 (1999) 91–102. https://doi.org/10.1016/S0376-7388(98)00243-9.
5. Damodar, R.A.; You, S.–J.; Chou, H.–H. Study the self cleaning, antibacterial and photo catalytic properties of TiO2 entrapped PVDF membranes, J. Hazard. Mater. 172 (2009) 1321–1328. https://doi.org/10.1016/j.jhazmat.2009.07.139.

6. Kang, G.; Yu, H.; Liu, Z.; Cao, Y. Surface modification of a commercial thin film composite polyamide reverse osmosis membrane by carbodiimide-induced grafting with poly(ethylene glycol) derivatives. Desalination 2011, 275, 252–259. https://doi.org/10.1016/j.desal.2011.03.007.
7. Bae, T.H.; Tak, T. M. Preparation of TiO_2 self-assembled polymeric nanocomposite membranes and examination of their fouling mitigation effects in a membrane bioreactor system, J. Membr. Sci. 266 (2005) 1–5. https://doi.org/10.1016/j.memsci.2005.08.014.
8. Choi, H.; Zhang, K.; Dionysiou, D.D.; Oerther, D.B.; Sorial, G.A.; Effect of permeate flux and tangential flow on membrane fouling for wastewater treatment. Sep. Purif. Technol. 45(2005)68–78. https://doi.org/10.1016/j.seppur.2005.02.010.
